# Digital arrest in the cyber age: a psychological perspective on fear, authority, and consciousness

**DOI:** 10.3389/fpsyg.2026.1726740

**Published:** 2026-03-04

**Authors:** S. James Robert, Varsha Singh, Ravi P. Pandey, Bismirty Bhuyan

**Affiliations:** 1 Department of Psychology, CHRIST University, Bangalore, Karnataka, India; 2 Department of Psychology, Central University of Haryana, Haryana, India; 3 Clinical Psychologist (RCI), Stepcare, Bangalore, India

**Keywords:** authority impersonation, compliance, criminology, cyber threat, cybercrime, cybersecurity, digital arrest, digital scam

## Abstract

Digital arrest is an emerging form of cyber deception wherein cybercriminals impersonate law enforcement or other authorities to falsely claim legal authority to arrest individuals through digital means, often via phone calls or online communication channels. As digital technology increasingly permeates daily life, such deceptive tactics pose serious threats to individuals' security and psychological well-being. This paper proposes a conceptual framework for understanding the phenomenon of “digital arrest”, differentiating it from other cybercrimes like phishing, vishing, and social engineering. The paper examines the psychological mechanisms underlying such scams, including the exploitation of fear, authority, and urgency, as well as the social implications of digital deception. Ultimately, the paper highlights the necessity for future research to empirically assess and evaluate the effectiveness of preventive measures and strategies aimed at reducing victimization. This conceptual paper aims to raise awareness of digital arrest as a distinct form of cyber threat and contribute to the growing body of literature on digital scams and their psychological consequences.

## Introduction

Cybersecurity threats are becoming more intricate and sophisticated in today's society, where the internet has become essential for social contact, employment, and communication (Mallick and Nath, 2024). One such cyber threat is *digital arrest*, a deceptive tactic increasingly employed by criminals. Criminals pose as law enforcement officials or other authorities in these frauds, claiming to have the legal right to make an arrest and frequently threatening to take legal action if they don't comply with demands for quick money payments or other cooperation. Indian Prime Minister Narendra Modi addressed this growing concern during his *Mann Ki Baat* radio program, where he urged people to stay vigilant. He highlighted the rise of cybercrimes and digital arrest scams in particular. Modi noted that criminals impersonate officials from police departments, CBI, Narcotics Enforcement Agency, and RBI to intimidate individuals and extort large sums of money. In an audio clip broadcast for awareness, the Prime Minister demonstrated how fraudsters might pretend to be police officers, requesting personal information like Aadhaar details to block a mobile number, urging people to take such warnings seriously and report any suspicious activity immediately. While the term digital arrest has yet to be widely recognized in the academic literature, this article seeks to define and contextualize the phenomenon, exploring its underlying psychological mechanisms, societal implications, and future research directions.

Recent trends in cybercrime reporting in India indicate a marked rise in frauds involving impersonation of law enforcement and regulatory authorities through phone and video calls. National cybercrime portals and media reports have repeatedly highlighted cases in which individuals are coerced into immediate compliance through threats of arrest, legal action, or asset seizure, often resulting in significant financial loss and psychological distress. While such incidents are increasingly visible in public discourse, they are frequently subsumed under broad categories such as phishing, vishing, or cyber extortion, limiting analytical attention to their distinct psychological dynamics. As a result, the authority-driven, fear-inducing mechanisms that characterize these scams remain insufficiently conceptualized within psychological scholarship. This gap necessitates a clearer definitional and theoretical examination of a specific phenomenon increasingly referred to as digital arrest.

## Review of literature

Although digital arrest is an emerging field of cybercrime, a limited but growing body of scholarly work has begun to examine its characteristics and implications. Existing studies have primarily focused digital arrest from legal, policy, and cybersecurity perspectives, with comparatively less attention to its psychological mechanisms.

Joseph (2026) studied a broad ethico-legal perspective on digital arrest, fraud, and identity theft, situating the topic within broader cybersecurity frameworks and highlighting legal gaps and AI-assisted decision support strategies. This work essentially contributed to understanding legal and technological defenses, especially in the context of automated detection and response systems, but it did not explicitly model the underlying psychological mechanisms that drive victim compliance or analyzed the fear-based process central to digital arrest. Similarly, Chauhan (2024) provided one of the earliest focused treatments of digital arrest as an emerging cybercrime in India, employing a descriptive approach to outline its core characteristics and socio-economic implications. While this study made an important early contribution by recognizing digital arrest as a discrete form of cybercrime, it remained primarily descriptive and lacked engagement with existing theoretical models of fear, authority influence, and compliance.

Furthermore, Singh and Singh (2024) critically examined the legality and practical feasibility of digital arrest warrants in India, highlighting the absence of statutory support for such practices and the disconnect between lawful arrest procedures and fraudulent impersonation tactics. While this legal analysis clarified institutional vulnerabilities, it did not engage with the psychological mechanisms that foster victim compliance in digital arrest scams. In addition, Sharma et al. (2026) examined technological advancements in Indian policing, including the evolving role of digital arrest mechanisms within broader smart policing initiatives. Although this study emphasized the use of digital technologies by law enforcement and the operational consequences for crime prevention and citizen involvement, it did not particularly examine the psychological mechanisms that drive victim involvement in cases of fraudulent digital arrests.

These contributions establish important groundwork, but there remains a need for deeper theoretical articulation and psychological contextualization of digital arrest as a distinct form of deception. The present study builds on and extends this emerging work by explicitly situating digital arrest within established psychological and behavioral theory, including social influence, obedience, and threat appraisal. By utilizing these frameworks, the current study not only emphasizes the distinctive characteristics of digital arrest but also clarifies the reasons behind and mechanisms by which particular cognitive and affective processes are triggered in victims, a topic that has not received enough attention in the body of existing literature.

## Recent trends and emerging significance of digital arrest scams

The growing incidence and seriousness of digital arrest in India are further highlighted by recent media outlets that use official cybercrime data. According to national newspapers, there was a sharp increase in such cases in 2024–2025. Cases were reported in numerous states, and victims suffered significant financial losses ranging from lakhs to crores (The Indian Express; The Times of India; Hindustan Times). These scams use phone and video calls to pose as law enforcement and regulatory bodies, predominantly targeting retired individuals and senior citizens. Rapid fear induction, perceived legal immediacy, and limited decision-making processes are common patterns in reported cases, which cause victims to comply with financial demands before verification is feasible. Unlike frauds such as dating or romance scams, which typically unfold over extended periods through emotional manipulation, digital arrest scams compress coercion into short, high-pressure interactions. The concentration of significant financial and psychological effects in short periods of time emphasizes digital arrest as a high-impact, fear-based cybercrime that demands intensive theoretical and psychological attention. Table 1 summarizes the aggregated trends and recurring characteristics of reported digital arrest scams in India during 2024–2025, based on nationally reported cybercrime cases.

**Table 1:** Aggregated trends in reported digital arrest scams in india (2024–2025).

**Indicator**	**Observed pattern**
Time frame of reported incidents	2024–2025
Geographic spread	Multiple Indian states including Karnataka, Maharashtra, Delhi, Telangana, Uttar Pradesh
Predominant victim profile	Older adults, retirees, and elderly individuals
Typical financial loss per case	Ranges from several lakhs to multiple crores
Highest reported individual loss	32 crore
Mode of impersonation	Police, CBI, NCB, TRAI, and other regulatory authorities
Communication channels used	Phone calls and video calls
Core psychological tactic	Fear of arrest, legal action, and asset seizure
Nature of compliance	Immediate financial transfers under perceived legal threat
Primary sources	The Indian Express, The Times of India, Hindustan Times, The Economic Times

## Definition of digital arrest

Digital arrest refers to a form of authority-impersonation cyber scam in which perpetrators, using real-time digital communication channels such as phone or video calls, falsely claim legal power to arrest the victim. The scam is designed to induce fear of imminent legal or physical detention, thereby compelling rapid compliance, typically in the form of financial payment or the disclosure of sensitive personal information. Central to this deception is the strategic use of urgency, intimidation, and institutional symbolism, which suppresses reflective judgment and accelerates obedience.

Unlike other forms of cybercrime such as phishing, vishing, smishing, or ransomware-based extortion, digital arrest does not rely on technical intrusion, data encryption, or the actual restriction of digital access. Traditional scams commonly impersonate banks or service providers to exploit curiosity, reward anticipation, or generalized fear of account compromise (Chebii, 2021; Yeboah-Boateng and Amanor, 2014). In contrast, digital arrest scams are distinguished by their real-time, interactive impersonation of law enforcement or legal authorities and by the invocation of arrest as an immediate threat. Their effectiveness lies not in technological control but in psychological coercion, particularly the manipulation of authority bias and fear of punishment.

## Bridging the gap: integrating digital arrest into cybercrime research

It is essential to comprehend the distinct features of digital arrest scam in order to develop specialized preventative strategies. Although phishing, malware, and ransomware are frequently highlighted in broader cybercrime research, these fraudulent activities primarily take into account authority and fear, necessitating targeted educational campaigns. Button et al. (2021) highlighted the severe psychological toll of scams rooted in authority abuse, which often cause both emotional trauma and financial damage. Tackling this niche form of cybercrime can enhance protective measures and strengthen confidence in digital platforms.

## The perpetrators

Perpetrators of digital arrest scams are typically organized cyber-fraud actors who specialize in authority impersonation rather than technical system intrusion. Unlike ransomware operators or malware-based extortionists, these individuals do not seek to restrict digital access, encrypt data, or exert prolonged technological control over victims. Instead, their primary tools are psychological scripts, symbolic authority cues, and real-time interpersonal interaction designed to elicit fear and immediate compliance. These perpetrators commonly impersonate officials from law enforcement agencies, regulatory bodies, or judicial institutions, adopting formal language, procedural terminology, and institutional symbolism to enhance perceived legitimacy. The use of caller ID spoofing, fake official identification, scripted legal accusations, and staged video-call environments resembling police offices further strengthens the illusion of authority. Such strategies exploit deeply ingrained social norms of obedience to legal authority, particularly in cultural contexts where law enforcement commands high respect.

The Perpetrators often operate in coordinated networks, with roles divided between callers, technical facilitators, and financial intermediaries. Their objective is short-term psychological domination rather than long-term surveillance or control. By creating a sense of imminent arrest or legal action, perpetrators narrow the victim's cognitive focus, suppress verification behaviors, and accelerate compliance. This reliance on emotional coercion distinguishes digital arrest scams from other cybercrimes that depend primarily on technological enforcement or data manipulation.

## The victims

Victims of digital arrest scams are not defined primarily by technical vulnerability or high digital exposure, but by psychological and situational susceptibility to authority-based deception. Individuals who exhibit high trust in institutional authority, low familiarity with legal procedures, or limited confidence in managing digital threats are particularly at risk. The effectiveness of digital arrest scams lies in their ability to exploit fear of legal consequences rather than deficiencies in technological safeguards. Older adults are frequently targeted due to limited experience with digitally mediated law enforcement interactions and a stronger tendency to defer to perceived authority figures. The formal language, legal terminology, and urgency employed by scammers can overwhelm their capacity for verification, leading to rapid compliance. Similarly, young adults and students may lack procedural knowledge of legal systems and are often unprepared for authority-impersonation tactics, making them vulnerable when confronted with sudden accusations and threats of arrest.

Working professionals and individuals managing personal or family finances are also susceptible, particularly when contacted during periods of stress, cognitive load, or time pressure. In such contexts, fear-induced narrowing of attention reduces critical evaluation and increases reliance on compliance as a threat-avoidance strategy. Importantly, victims of digital arrest are often psychologically unprepared for real-time confrontation with fabricated legal authority, regardless of their general digital literacy or educational background. Hence, vulnerability to digital arrest is best understood as a function of emotional reactivity, authority bias, and reduced self-efficacy under perceived legal threat, rather than as a consequence of technical exposure or high-value digital assets. Recognizing this distinction is essential for developing psychologically informed prevention and intervention strategies.

## Integrating prior research: psychological mechanisms in cyber deception

Existing research in cyberpsychology and the psychology of deception provides valuable insights into how fear, perceived authority, and digital literacy shape vulnerability to online manipulation. Studies on phishing and social engineering have highlighted that scammers often use fear appeals and authority cues to trigger compliance (Workman, 2008; Riek et al., 2015). Victims frequently react impulsively under pressure, especially when confronted with threats framed as urgent or legitimate. This dynamic parallels the mechanisms underlying digital arrest, where fear of punishment and respect for authority drive immediate obedience. Together, these findings establish a foundational psychological framework for understanding authority-based deception in digital environments.

Research by Vishwanath et al. (2018) and Muhanad et al. (2025) further underscores that low digital literacy and excessive trust in authority figures increase susceptibility to such manipulative tactics. Similarly, studies by Kemp and Erades Pérez (2023) and Norris and Brookes (2021) revealed that exposure to internet fraud is increased by psychological vulnerability, especially in elderly persons and novice users. These findings point to the psychosocial dimensions of cyber victimization, where emotional triggers outweigh rational decision-making.

Moreover, knowledge of cybersecurity has been repeatedly found to be a crucial protective factor by researchers. People who are more knowledgeable about online threats and safety precautions are less susceptible to deception (Zwilling et al., 2020; Verkijika, 2019; Das and Patel, 2017; Asher and Gonzalez, 2015). Nevertheless, despite this growing body of research, fear-based cybercrimes that explicitly imitate legal authority and threaten imminent arrest remain largely under-theorized in the literature; the concept of digital arrest seeks to address this gap. Placing this phenomenon in the larger context of cyber deception broadens the scope of psychological research from risky behavior in general to the complex interactions between fear, obedience, and trust in digital environments.

## Mechanisms and stages of digital arrest

To move beyond a descriptive account of digital arrest and toward an explanatory framework, the present analysis is anchored in two complementary psychological perspectives: obedience to authority and Technology Threat Avoidance Theory (TTAT). Obedience-based models explain why individuals comply with perceived legal authority even in the absence of physical coercion, while TTAT elucidates how perceived threat severity, susceptibility, and self-efficacy shape individuals' responses to digitally mediated threats. Together, these lenses provide a coherent theoretical foundation for understanding how authority impersonation, fear induction, and compliance unfold across the three stages of digital arrest described below.

### Stage 1: Initiation

During the initiation stage, perpetrators establish contact through real-time digital communication channels such as phone calls or video interactions, deliberately impersonating law enforcement or other legal authorities. Rather than relying on physical presence, scammers construct symbolic authority through formal language, procedural terminology, institutional references, and genuine logos. These cues function as legitimacy signals that activate automatic respect for authority in the recipient.

This process can be analytically understood through Milgram's (1963) theory of obedience, which demonstrated that compliance arises not solely from physical coercion but from the perception of legitimate authority. In digitally mediated contexts, authority is disembodied yet psychologically potent, as individuals increasingly interpret institutional symbols and communication protocols as markers of authenticity. The absence of physical presence does not diminish obedience; rather, digital impersonation reframes authority into a symbolic and procedural form that discourages skepticism and delays verification. Consequently, the initiation stage establishes the psychological conditions necessary for subsequent fear-based manipulation by normalizing the scammer's authority before any explicit threat is introduced.

### Stage 2: Manipulation

Once the initial trust has been established, scammers use urgency and anxiety to intensify the emotional response. This stage can be systematically explained through Technology Threat Avoidance Theory (TTAT), which posits that individuals' responses to technological threats are shaped by perceived severity, perceived susceptibility, and coping appraisals (Chen and Liang, 2019). In digital arrest scams, perceived severity is amplified through references to criminal charges and arrest, while perceived susceptibility is reinforced by personalized accusations involving identity documents or financial records. Simultaneously, victims' self-efficacy is undermined by legal complexity and time pressure, reducing confidence in their ability to respond effectively.

Under these conditions, fear-driven threat appraisal outweighs rational verification processes, and compliance emerges as a maladaptive coping response aimed at immediate threat reduction (Furedi, 2018). While cognitive dissonance may arise between the individual's belief in their innocence and the authority's allegations (Festinger, 1957a,b), the urgency of the situation prioritizes emotional relief over logical consistency. Thus, the manipulation stage represents the critical transition point at which authority-induced fear, as conceptualized by TTAT, reshapes decision-making toward rapid obedience.

### Stage 3: Compliance

In the final stage, victims engage in behaviors aimed at immediately neutralizing the perceived threat, such as transferring money, disclosing sensitive information, or maintaining prolonged interaction with the impersonator. These actions should not be understood as reckless but as psychologically adaptive responses within a context of acute fear and constrained choice.

From the perspective of obedience to authority, compliance emerges when the perceived legitimacy and power of the authority figure override personal judgment (Milgram, 1963; Kelman, 1958). In digitally mediated encounters, the combination of symbolic authority cues and escalating threat reinforces the belief that obedience is the safest course of action. Simultaneously, as articulated by Technology Threat Avoidance Theory (TTAT), compliance functions as a maladaptive coping strategy, wherein individuals prioritize immediate threat reduction over long-term risk evaluation due to diminished self-efficacy and heightened perceived severity.

**Figure 1:**
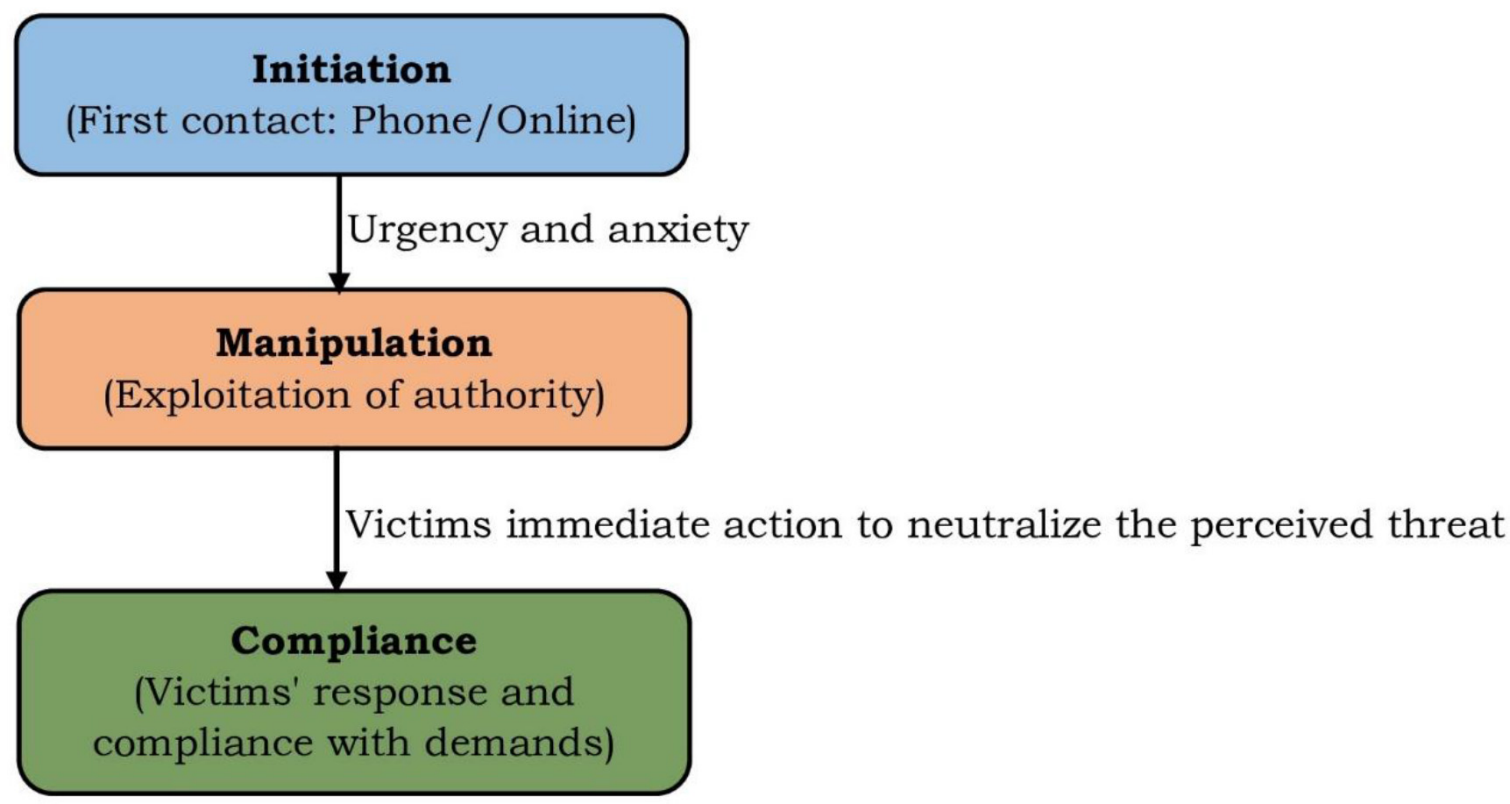
Stages of digital arrest.

Cognitive functioning under such stress is further compromised, increasing susceptibility to impulsive decision-making and narrowing attention to short-term relief (Starcke and Brand, 2012). Fear of legal consequences, social exposure, or reputational damage reinforces obedience, making disengagement psychologically costly. Thus, the compliance stage represents the culmination of authority-based coercion and threat-driven avoidance, where obedience and maladaptive coping converge to produce victimization. Figure 1 illustrates the sequential psychological stages of digital arrest scams, highlighting the conditions that trigger progression from authority recognition to fear-induced compliance.

Across the three stages of digital arrest, victimization is shaped by the interaction of authority-induced fear, perceived urgency, and automatic compliance. Scammers deliberately orchestrate these psychological processes, a strategy consistent with Cialdini (2009) principles of persuasion, which emphasize the heightened influence of authority cues and time pressure on decision-making. Fear of legal consequences, in particular, activates threat-avoidance responses that suppress reflective evaluation and narrow attention to immediate relief. Accordingly, digital arrest is best understood not as a technologically sophisticated hoax but as a psychologically structured process that manipulates emotion, cognition, and consciousness through digitally mediated interactions. Recognizing this processual nature is essential for developing preventive interventions that enhance digital awareness, emotional regulation, and psychological resilience rather than focusing solely on technical safeguards.

## Preventive perspectives and future directions

Building on the obedience- and threat-avoidance–based mechanisms identified in the preceding stages, effective prevention of digital arrest scams must address not only technological vulnerabilities but also the psychological processes that drive authority-based compliance and threat-driven decision-making. Recognizing that these scams operate by manipulating consciousness rather than technology alone reframes prevention as a matter of both cognitive resilience and digital empowerment. While awareness campaigns remain central to prevention, their effectiveness depends on whether they directly target the authority bias and fear-induced urgency that characterize digital arrest scams. These programs must, however, address the psychological aspects of deception in addition to technical guidance. Enhancing users' self-efficacy through targeted education not only about avoiding suspicious links or calls but also about recognizing the emotional cues including fear, urgency, and authority pressure that scammers exploit. By incorporating these psychological principles into digital literacy programs—such as training individuals to recognize the typical emotional trajectory from authority invocation to escalating urgency—users can develop emotional readiness that enables them to pause, question, and verify before reacting impulsively.

Cybersecurity and law enforcement organizations are also very important. Specialized training focused on authority-impersonation dynamics and fear-based compliance can improve officials' ability to recognize and respond to manipulation-driven frauds such as digital arrest. Collaboration between law enforcement, psychologists, and communication experts can lead to the design of evidence-based educational modules and simulated training environments that mirror real scam interactions. Such psychologically informed simulations may function as cognitive inoculation tools, reducing susceptibility to authority-based coercion by familiarizing individuals with the emotional and behavioral patterns characteristic of digital arrest scams.

From a complementary technical perspective, online portals and telecommunication companies can incorporate artificial intelligence (AI)-powered detection systems that highlight potentially fraudulent communications, such as impersonations of authority figures. Verified digital credentials or caller ID authentication for government and law enforcement communications could further reduce the effectiveness of impersonation tactics.

From a theoretical perspective, Technology Threat Avoidance Theory (TTAT) offers a valuable framework for understanding the way individuals perceive, evaluate, and act upon cyber threats. As proposed by Chen and Liang (2019), individuals' motivation to avoid threats depends on their perceived susceptibility, severity, self-efficacy, and belief in safeguard effectiveness. By applying this framework to analyze digital arrest, it is possible to decrease compliance with scammers by raising users‘ self-efficacy; their confidence in managing digital threats; and raising their knowledge of the seriousness of manipulative tactics.

For future research, integrating TTAT with models from cyberpsychology and behavioral economics could help explain why individuals under stress prioritize immediate relief over rational verification. The relationship between trust, cognitive load, and digital exhaustion and vulnerability to authority-based scams could potentially be investigated further. Such investigations can lead to interventions that strengthen both psychological and technological readiness. In the end, a thorough preventative approach needs to foster digital consciousness, or a critical understanding of the ways in which technology, human emotion, and cognition interact. Strengthening such digital consciousness can reduce susceptibility to authority-based, fear-driven coercion such as digital arrest, while also supporting more reflective and resilient engagement with digitally mediated environments.

## Implications for consciousness and society

Beyond individual victimization, digital arrest scams have far-reaching implications for human consciousness in the digital era. These deceptions blur the boundaries between technological interaction and psychological experience, shaping how people perceive authority, trust, and safety in virtual environments. Every experience with such a scam triggers both cognitive processing and ingrained emotional reactions associated with fear and compliance, illustrating how digital communication is currently functioning as a psychological influence medium. Repeated exposure to fraudulent authority cues may contribute to reduced trust in digital communication and increase skepticism toward legitimate institutions. Individuals may become more cautious or skeptical of genuine outreach from banks, government agencies, or law enforcement, which could pose challenges for effective digital governance. This phenomenon reflects a growing tension in digital life, wherein users increasingly rely on technology for convenience while simultaneously fearing its potential for manipulation. Over time, this tension can diminish users' confidence in both their digital literacy and their ability to discern authenticity online.

At a psychological level, the experience of digital arrest often produces emotional distress, anxiety, and shame. Victims may feel violated or humiliated after realizing they were deceived, leading to self-blame and social withdrawal. These emotional consequences align with research on fear-based persuasion, which shows that intense fear coupled with perceived helplessness can reduce psychological wellbeing and decision-making capacity.

From a societal standpoint, widespread incidents of authority-impersonation scams such as digital arrest may influence public perceptions of trust in digital infrastructure, slowing down adoption of digital services and discouraging participation in online transactions. When authority figures are impersonated digitally, perceptions of institutional legitimacy may be challenged, particularly in digitally mediated interactions. This creates a paradox where the same technologies designed to increase accessibility and efficiency become channels for deception and distrust. Ultimately, digital arrest illustrates how psychological vulnerability can shape conscious experience and decision-making in digitally mediated contexts. It reveals the urgent need to strengthen not only technological defenses but also psychological resilience and critical digital awareness, ensuring individuals can navigate the online world with informed vigilance rather than reactive fear.

## Conclusion

In the digital era, the phenomenon of “digital arrest” highlights how fear, authority, and technology may all work together to manipulate people's perceptions. It represents a deeper psychological manipulation than simple online fraud, involving the use of emotional susceptibility, cognitive shortcuts, and obedience to perceived authority. Understanding this form of cyber deception thus requires an integrative lens that bridges psychology, communication, technology, and social behavior.

As daily life becomes more and more mediated by digital contacts, it is essential to develop digital consciousness, or the understanding of how technology interacts with emotion and consciousness. Efforts to prevent such scams must move beyond technical defenses to address the psychological and societal dimensions that sustain them. Collaboration among behavioral scientists, law enforcement, educators, and technologists can foster resilience through critical awareness, empathy, and trust. Ultimately, recognizing digital arrest as a phenomenon rooted in psychology and consciousness advocates a more compassionate and proactive approach to digital safety; one that empowers individuals not through fear, but through understanding.

People should be cautious of unsolicited calls, particularly if the caller poses as an official and demands personal information or payment. Cybercriminals often use pressure tactics, creating a false sense of urgency to prompt quick decisions. People should avoid giving important information over the phone, such as banking or identity credentials, and should confirm the caller's authenticity by getting in touch with the organization directly. People should use only official channels for communication, as government agencies do not use platforms like WhatsApp or Skype for official matters. If they suspect a scam, they must report it to local police or cybercrime authorities.

## Data Availability

The original contributions presented in the study are included in the article/supplementary material, further inquiries can be directed to the corresponding author.
